# RNA-Seq-based transcriptional map of the bovine respiratory disease pathogen *Histophilus somni* 2336

**DOI:** 10.1186/gb-2011-12-s1-p40

**Published:** 2011-09-19

**Authors:** Ranjit Kumar, Mark L Lawrence, James Watt, Amanda Cooksey, Shane C Burgess, Bindu Nanduri

**Affiliations:** 1 College of Veterinary Medicine, Mississippi State University, Mississippi State, MS 39762, USA; 2 Institute for Genomics, Biocomputing and Biotechnology, Mississippi State University, Mississippi State, MS 39762, USA; 3 Center of Biostatistics and Bioinformatics, University of Mississippi Medical Center, Jackson, MS 39216, USA; 4 Eagle Applied Sciences, Clinical Research Laboratory, 81st Medical Group, Keesler AFB, MS, USA

## Background

Genome structural annotation - that is, the identification and demarcation of the boundaries of all of the functional elements in a genome (such as the genes, non-coding RNAs, proteins and regulatory elements) - is a prerequisite for systems level analysis. Current genome annotation programs do not identify all of the functional elements of the genome, especially small non-coding RNAs (sRNAs). Transcriptome analysis is a complementary method for identifying ‘novel’ genes, small RNAs, regulatory regions and operon structures, thus improving structural annotation in bacteria. In particular, the identification of non-coding RNAs has revealed their widespread occurrence and functional importance in gene regulation, stress and virulence. However, very little is known about non-coding transcripts in *Histophilus somni*, one of the causative agents of bovine respiratory disease, as well as bovine infertility, abortion, septicemia, arthritis, myocarditis and thrombotic meningoencephalitis.

## Methods

In this study, we generated a single-nucleotide resolution transcriptome map of *H. somni* strain 2336 using RNA-Seq (Illumina). A Perl script was written to convert Illumina reads into FASTQ format. The software tools MAQ, Bowtie and SAMtools were used to process the raw data and generate pileup format, which provides the signal map file in per-base format coverage. In-house Perl scripts were written to identify novel sRNAs, putative novel proteins and operon structures. Comparative genomic analysis of *H. somni* strain 2336 and the avirulent strain 129Pt was performed using the tool Mauve. The processed data were submitted to the Gene Expression Omnibus database with accession number GSE29578.

## Results

The RNA-Seq-based transcriptome map identified 94 sRNAs in the *H. somni* genome, of which 82 had not been predicted or reported in earlier studies. We also identified 38 novel potential protein-coding ORFs that are not in the current genome annotation. The transcriptome map allowed the identification of 278 operon structures (for a total of 730 genes) in the genome. Compared with the genome sequence of a non-virulent strain, 129Pt, a disproportionate number of sRNAs (about 30%) were located in a genomic region unique to strain 2336 (accounting for about 18% of the total genome). This observation suggests that a number of the newly identified sRNAs in strain 2336 may be involved in strain-specific adaptations that could include virulence.

## Conclusions

Overall, this study describes an RNA-Seq-based transcriptome map of *H. somni*, an important agricultural pathogen, that was constructed to identify functional genomic elements. Our genome-wide survey predicts numerous novel expressed regions that need to be characterized biologically to improve our understanding of disease pathogenesis. A description of all of the functional elements in the *H. somni* system is a prerequisite for using holistic systems approaches to understand the complex pathogenesis of bovine respiratory disease.

